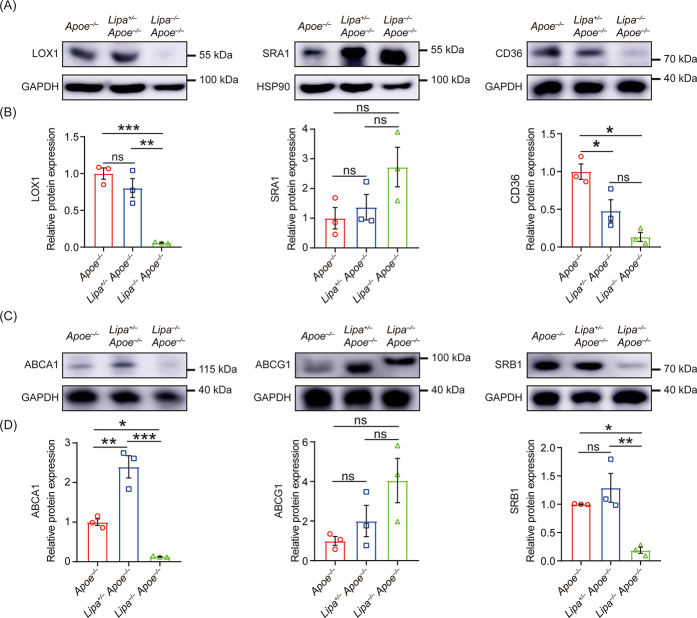# *Lipa* regulates myeloid differentiation and is essential for intra-plaque macrophage accumulation during atherogenesis

**DOI:** 10.1016/j.jlr.2026.101092

**Published:** 2026-06-29

**Authors:** Tianhan Li, Juanjuan Qiu, Haoyue Zhang, Jianjun Wu, Xiaoyu Wang, Jing Wen, Xin Dong, Rui Fu, Liaoxun Lu, Lichen Zhang, Toby Lawrence, Marc Bajenoff, Yinming Liang, Rong Huang, Zhongshu Liang

**Affiliations:** 1 School of Basic Medical Medicine, Henan Medical University, Xinxiang, China; 2 Laboratory of Genetic Regulators in the Immune System, School of Medical Technology, Henan Medical University, Xinxiang, China; 3 Centre for Inflammation Biology and Cancer Immunology, Cancer Research UK King’s Health Partners Centre, School of Immunology and Microbial Sciences, King’s College London, London, UK; 4 Aix Marseille Univ, CNRS, INSERM, CIML, Marseille, France; 5 Center of Disease Model and Immunology, Hunan Academy of Chinese Medicine, Changsha, China; 6 Department of Cardiology, Xiangya third hospital, Central south university, Changsha, China

**Keywords:** LIPA, atherosclerosis, macrophages, foam cell formation, CD36

## Abstract

Lysosomal acid lipase (*LIPA*), one of the earliest and most impactful genetic factors linked to human coronary artery diseases, is highly expressed in macrophages despite predominant hepatic production. However, the functional importance of *LIPA* in macrophages remained largely unknown. Notably, individuals with atherosclerosis-risk alleles demonstrate elevated *LIPA* expression in monocytes and macrophages, but lower levels in liver and plasma, indicating a potential macrophage-specific regulatory role of *LIPA* on atherosclerosis. The development of atherosclerosis in genetic models lacking *LIPA* has presented challenges. To investigate whether macrophage LIPA influences atherosclerosis, we established *Lipa*-deficient mice on an *Apoe*^*−/−*^ background. *Lipa*^*−/−*^*Apoe*^*−/−*^ mice developed hepatosplenomegaly and enhanced myelopoiesis after being fed a high-fat diet, which aligns with observations in human *Lipa* deficiency. Unexpectedly, both *Lipa*^+/−^*Apoe*^*−/−*^ and *Lipa*^*−/−*^*Apoe*^*−/−*^ mice showed significantly attenuated atherosclerosis. This protection was recapitulated in *Apoe*^*−/−*^ recipients reconstituted with bone marrow from either *Lipa*^+/−^*Apoe*^*−/−*^ or *Lipa*^*−/−*^*Apoe*^*−/−*^ donors. Mechanistically, such atheroprotection was linked to a notable reduction in foam cell formation and macrophage accumulation within plaques. Homozygous deficiency suppressed foam cells via impaired monocyte development and CD36 downregulation, whereas heterozygosity primarily decreased CD36 expression. *In vitro*, *Lipa*^*−/−*^ macrophages exhibited diminished lipid uptake and CD36 expression, both reversed by *Lipa* re-expression. Notably, reduced *Lipa* expression in *Lipa*^+/−^*Apoe*^*−/−*^ mice did not impact hepatic lipid metabolism or macrophage differentiation, as evidenced by flow cytometry and single-cell RNA sequencing. These findings highlight a novel role for *Lipa* in modulating macrophage behavior during atherosclerosis, suggesting that *Lipa* may serve as a promising therapeutic target for the treatment of atherosclerosis.

Lysosomal acid lipase (LIPA) is the key enzyme responsible for hydrolyzing cholesteryl esters (CEs) and triglycerides (TGs), which are taken up through the endocytosis of lipoproteins in the lysosomal compartment (1, 2). Mutations in the *LIPA* gene result in rare lysosomal lipid storage disorders characterized by a complete or partial absence of LIPA activity, typically leading to liver dysfunction (3). Individuals with complete functional loss of LIPA often experience severe morbidity and mortality, with the phenotypic spectrum ranging from the infantile form (Wolman's disease, WD) to the delayed form collectively known as cholesterol ester storage disease (CESD) (4). Previous reports have indicated that patients with CESD often suffer from dyslipidemia, premature atherosclerosis, and early cardiovascular events (5, 6). However, the relationship between the development of cardiovascular disease as a secondary consequence of dyslipidemia and the disruption of lipid and immune homeostasis leading to atherogenesis, remains poorly understood (3). Genome-wide association studies (GWAS) have identified *LIPA* as a risk locus for cardiovascular disease (7, 8, 9). Interestingly, risk alleles were not associated with reduced expression of LIPA in the liver or plasma lipid levels (8), but paradoxically with higher *LIPA* mRNA in monocytes and macrophages (10, 11). The potential impact of this increased *LIPA* *mRNA* expression and enzymatic activity, and its potential role in exacerbating atherosclerosis, remains a subject of controversy.

Atherosclerosis is a chronic inflammatory vascular disease and the predominant cause of cardiovascular diseases, resulting in substantial morbidity and mortality on a global scale (12, 13, 14). The critical progression of atherosclerosis, whether in humans or mice, is the formation of foam cells (15, 16). The accumulation of excessive subendothelial modified lipids in macrophages leads to their differentiation into foam cells (17), which are abundant within atherosclerotic plaques and accelerate the development of atherosclerotic lesions (18, 19). Recent studies have suggested that extracellular LIPA may enhance the lipid uptake of macrophages in diseased arteries, thereby leading to foam cell formation (18, 19). This finding appears contradictory to observations in animal models and CESD patients, where the loss of LIPA causes plaque macrophage dysfunction and atherosclerosis. Therefore, it is crucial to thoroughly understand the full range of LIPA functions in intraplaque macrophages to comprehend the development of atherosclerosis.

In this study, we investigated the role of *Lipa* in atherosclerosis using *Lipa* knockout mice on an *Apoe*^*−/−*^ background. Our analysis revealed that *Lipa*, with 95% sequence homology between mouse and human (20), plays a significant role in atherosclerosis. Through the use of knockout and knock-in macrophage lines, along with *Lipa-deficient* animal models, we demonstrated that *Lipa* deficiency contributes to atheroprotection. Our findings were from high-fat diet-treated *Lipa*^*−/−*^ *Apoe*^*−/−*^ mice mimicking CESD patient symptoms. Additionally, single-cell RNA sequencing revealed a notable increase in splenic neutrophils and macrophages in *Lipa*^*−/−*^ *Apoe*^*−/−*^ mice. Notably, both *Lipa*^*+/−*^ *Apoe*^*−/−*^ mice and *Lipa*^*−/−*^ *Apoe*^*−/−*^ mice exhibited a remarkable reduction in the severity of atherosclerosis, accompanied by a reduction in necrotic core area and macrophage accumulation within plaques. This athero-protective phenotype was recapitulated in *Apoe*^*−/−*^ recipients reconstituted with Lipa-deficient bone marrow. Mechanistically, homozygous *Lipa* deficiency conferred plaque protection primarily by reducing foam cell formation via inhibition of monocyte development and CD36 downregulation. In contrast, heterozygosity was associated mainly with CD36 downregulation within plaques. Further in vitro experiments confirmed that *Lipa* deficiency impaired lipid uptake and suppressed CD36 levels, and these effects were reversed upon *Lipa* reconstitution. Unexpectedly, reduced *Lipa* expression in *Lipa*^*+/−*^ *Apoe*^*−/−*^ mice affected foam cell formation and macrophage accumulation, without impacting hepatic lipid metabolism. Our experiments using genetic models revealed the novel roles of *Lipa* in myeloid cell differentiation and CD36-mediated intra-plaque macrophage accumulation during atherosclerosis, which resolved previous controversies.

## Materials and Methods

### Animals

5- to 6-week-old *Apoe*^*−/−*^ C57BL/6 mice were purchased from Beijing Vital River Laboratory Animal Technology Co., Ltd. *Lipa*^*−/−*^ mice were constructed according to our previous study (21). Briefly, Cas9 mRNA and sgRNA were microinjected into the fertilized eggs from *Apoe*^*−/−*^ C57BL/6 mice, then cultured in M2 medium for 24 h to two-cell stage and finally transferred into femal ICR foster mother mice in the next day. *Apoe*^*−/−*^, *Lipa*^+/−^ *Apoe*^*−/−*^ mice and *Lipa*^*−/−*^ *Apoe*^*−/−*^ mice were generated by self-crossing *Lipa*^+/−^ *Apoe*^*−/−*^ mice. 7-8-week-old male *Apoe*^*−/−*^, *Lipa*^*+/−*^ *Apoe*^*−/−*^ and *Lipa*^*−/−*^ *Apoe*^*−/−*^mice were fed a high-fat diet (D12108 C with 40% fat and 1.25% cholesterol, purchased from Research Diets, Inc.) for 12 weeks. The body weight of each mouse was measured weekly. The weight of the spleen and liver of each mouse was measured when sacrificed. All animal procedures were performed according to guidelines approved by the committee on animal care at Henan Medical University.

### Single cell RNA sequencing of spleen

Single-cell suspensions from spleens were first stained with antibody CD45.2-PE and BD™ Ms Single Cell Sample Multiplexing Kit (BD Bioscience, Cat: 633793). After washing with 1 ml FACS buffer two times, cells were resuspended with Sytox blue and CD45.2^+^CD5^-^CD19^-^Ly6G^-^ cells were sorted and subjected to the BD Rhapsody Express system. Then cDNA and sample tag libraries were built with BD Rhapsody whole transcriptome amplification (WTA) reagent kit (BD Bioscience, Cat: 633733, 633,773, 633,801, 664,887) following manufacturer’s instructions and sequenced on an illumine Novaseq 600 sequencer. Pair-end Fastq files of sample tag and WTA data were processed via the BD Rhapsody™ analysis pipline v2.0, and the resultant dataset was mainly analyzed using SeGeq™ software, which contains Lex-BDSMK and Seurat v4.04 plugin components. Raw data and processed data were uploaded into GEO (Accession number: GSE273731).

### Bone marrow transplantation

Following one week of antibiotic treatment, recipient CD45.1^+^ Apoe^−/−^ mice received intraperitoneal injections of busulfan (30 μg/g body weight) every two days over the following week. Subsequently, bone marrow cells isolated from 7-8-week-old donor mice (CD45.2^+^ *Apoe*^*−/−*^, CD45.2^+^ *Lipa*^*+/−*^ *Apoe*^*−/−*^ mice and CD45.2^+^ *Lipa*^*−/−*^*Apoe*^*−/−*^) were intravenously transplanted into the recipient mouse at a dose of two million cells per mouse. Four weeks after transplantation, engraftment efficiency was assessed. All mice were then fed a HFD for 12 weeks. Finally, mice were sacrificed for analysis, which included *en face* Oil Red O (ORO) of the descending aorta, hematoxylin and eosin (H&E) staining, ORO staining and immunohistochemistry (IHC) of the aortic root, as well as flow cytometry analysis, as previously described (21).

### Atherosclerotic lesion analysis

After being fed with 12 weeks high-fat diet, each mice’s blood was obtained to check the level of total cholesterol (TC), triglyceride (TG), low-density lipoprotein (LDL), and high density lipoprotein (HDL) via an automated analyser platform (Roche Cobas C501, Roche Co., Switzerland), and the concentrations of free cholesterol, cholesteryl ester, triglyceride and phospholipid in different lipoproteins, including very low density lipoprotein (VLDL), intermediate density lipoprotein (IDL), LDL and HDL, were detected by Hefei Guangce Product Testing Institute. Then mice were perfused with heparinized saline, and heart, liver and the entire aorta were removed. En face descending aortas were fixed with 4% paraformaldehyde and then stained with Oil Red O (ORO). Frozen section of aortic root and liver were stained with hematoxylin and eosin (HE) and ORO for lesion quantification, and aortic root sections were perfomed with immunohistochemical analyses according to previous study (22) (Anti-CD68 Rabbit pAb: GB113109). Images were acquired on CaseViewer.

### Immunofluorescence and immunohistochemical analysis

Paraffin sections of the aortic root were dewaxed using a dewaxing solution, rehydrated through a graded ethanol series, and washed with distilled water. Antigen retrieval was performed under optimized conditions, and sections were allowed to cool naturally. After washing with PBS, endogenous peroxidase activity was blocked with 3% H_2_O_2_, followed by serum blocking to reduce nonspecific binding. Sections were incubated with primary antibodies (F4/80, GB113373; CD68, GB153109; CD36, GB113319) at 4°C overnight, followed by incubation with secondary antibodies. For immunohistochemistry, sections were stained with DAB at room temperature. Subsequently, they were counterstained with hematoxylin, dehydrated in graded ethanol, and mounted with neutral balsam. For immunofluorescence, nuclei were counterstained with DAPI, and autofluorescence was quenched before mounting with an antifade mounting medium. Images were acquired using an LG-FS80 slide scanner (Servicebio) and analyzed using Pannoramic MIDI software (3DHISTECH).

### Cell culture

RAW264.7 macrophages (purchased from the National Collection of Authenticated Cell Cultures) were cultured in DMEM (Dulbecco’s Modified Eagle’s Medium) containing 10% FBS (fetal bovine serum), 100 units/ml penicillin and 100 μg/ml streptomycin. For isolation of peritoneal macrophages, age- and sex-matched *Lipa*^*−/−*^ *Apoe*^*−/−*^ and *Apoe*^*−/−*^ mice were injected i.p. with 1.5 ml of 4% sterile thioglycolate brewer broth (BD). Three days after the injection, cells were harvested by *i.p.* lavage with ice-cold PBS. Then cells were seeded in DMEM medium with 10% FBS.

### CRISPR/Cas9-mediated genome editing of RAW264.7 macrophages

To generate gene-specific deletion in RAW264.7 macrophages via CRISPR/Cas9 system, three sgRNAs were designed for *Lipa* gene and individually cloned into the CRISPR-expressing vector pX458-DsReds or its derivative with ECFP reporter, allowing for single-cell sorting as described previously (21, 23).

For the generation of a gene knock-in at the safe harbor locus *Rosa26*, the mouse *Lipa* coding sequence (ENSMUST00000049572.15) with an N-terminal FLAG tag and a C-terminal OST tag was synthesized (Bioligo Biotechnology, Shanghai, China), and subsequently cloned into the targeting vector pKR26-iBFP using NEBuilder HiFi DNA Assembly Master Mix (New-England Biolabs). The resulting plasmid (C-3 clone) served as the template for homologous recombination to create a knock-in cell expressing FLAG-*Lipa*-OST along with a BFP (blue fluorescent protein) fluorescent reporter in *Lipa*^*−/−*^ RAW264.7 macrophages (KI). The constructs were transfected into RAW264.7 macrophages using the Neon® Transfection System (Thermo Fisher Scientific), following the protocol described in previous studies (24, 25). The deletions of the *Lipa* gene in wild-type (WT) RAW246.7 cells and the knock-in of FLAG-Lipa-OST in *Lipa*^*−/−*^ cells (KI) in were confirmed through PCR and sequencing analyses.

### Quantitative Real-Time PCR

The liver tissue (about 30 mg) was homogenized using RNase-free ceramic beads (Servicebio biotechnology Co.) following standard procedures of mechanical tissue disruption via tissue grinder (Servicebio biotechnology Co., Ltd). The total RNA was isolated using the RNeasy Plus Mini Kit (Qiagen). Reverse transcription was performed using RevertAid First Strand cDNA Synthesis Kit (Thermo Fisher Scientific). The purity and quantity of RNA and cDNA were analyzed using NanoDrop (PeqLab). The expression level of *Lipa* was quantitatively analyzed by qRT-PCR based on TB Green Premix Ex Taq™ (TaKaRa) using the Applied Biosystems ABI 7500. The primer sequences for the qRT-PCR were listed in supplemental Table S1.

### Detection of DiI-oxLDL uptake by flow cytometry

Unstained cells served as negative controls. RAW264.7 or peritoneal macrophages were seeded in 12-well plates and cultured for 24 h, followed by synchronization in DMEM supplemented with 0.5% FBS for 2 h. Cells were then incubated for 2 hours with DMEM containing 2% FBS and 50 μg/ml DiI-labeled oxLDL (YB-0010; Yiyuan Biotech). Macrophages from WT or *Apoe*^*−/−*^ mice without DiI-oxLDL incubation were used as fluorescence-minus-one (FMO) controls. After incubation, cells were collected, washed twice with ice-cold PBS, and resuspended in FACS buffer. Samples were acquired on a BD FACS Canto™ II flow cytometer, and data were analyzed using FlowJo™ Software (v10.1).

### Foam cell formation assay

Peritoneal macrophages were seeded into a 12-well plate and cultured for 24 h. Then cells were synchronized with DMEM containing 0.5% FBS for 6 h and incubated with 100 μg/ml ox-LDL (Yiyuan Biotech, Cat# YB-002; prepared by oxidizing human LDL with Cu_2_SO_4_ in PBS at 37 °C for 18 h, terminated by EDTA, and verified by agarose gel electrophoresis showing 2.0× migration vs. native LDL and TBARS with MDA >18.5 nmol/mg protein) for 24 h. After that, cells were fixed with 4% paraformaldehyde for 30 min and incubated with 85% 1, 2-propanediol for 2 min. Then the cells were stained with 0.3% ORO in isopropanol for 5 min. After washing with 85% 1, 2-propanediol and PBS, imaging was performed using a Nikon Eclipse Ts2 microscope.

### Flow cytometry analysis

To obtain single-cell suspension, aorta, spleen, femur and tibia were collected from each mouse. Then spleens were dissociated using the Gentle MACS Dissociators (Miltenyi Biotec) and aorta were acquired by mechanical disruption, and followed by digestion using digestive enzymes (Collagenase IV, Cat: V900893 from Sigma; DNase I, Cat:10104259001 from Sigma) for 30 min. Femur and tibia ends were clipped with sterile scissors and marrow cavities were flushed with 5 ml ice-cold PBS using a 25-gauge needle attached to a 5-mL syringe. Then, cells were filtrated with 75 μm strainers and stained with monoclonal antibody mixes after blocking with Fc receptor blocking agents. Cells were acquired by flow cytometers (Thermo Fisher Scientific, Invitrogen Attune NxT Flow Cytometer). Spleen neutrophils were gated on CD45 and Ly6G positive cells; B cells were gated on CD45 and CD19 positive cells; T cells were gated on CD45 and CD5 positive cells; RPM (red pulp macrophages) were gated on lin (CD3, CD19, Ly6G and NK1.1), F4/80 negative and CD11b positive cells; WPM (white pulp macrophages) were gated on lin (CD3, CD19, Ly6G and NK1.1), CD11b negative and F4/80 positive cells. Aorta macrophages were gated on CD3, CD19 and Ly6G negative, CD64 and F4/80 positive cells. Blood monocytes from three groups were gated on CD3, CD19 and Ly6G negative, CD115 and CD11b positive cells, which were further divided into Ly6C^high^ and Ly6C^low^ monocytes. Common myeloid progenitors (CMPs) from bone marrow were gated on CD45^+^CD16/32^lo^ CD117^+^CD34^+^CD135^+^ and Ly6C^-^CD115^-^. Monocyte-dendritic cell progenitors (MDPs) were gated on CD45^+^CD16/32^lo^ CD117^+^CD34^+^CD135^+^ and Ly6C^-^CD115^+^. Granulocyte-monocyte progenitors (GMPs) from bone marrow were gated on CD45^+^CD16/32^hi^ CD117^+^CD34^+^CD135^-^ and Ly6C^-^CD115^-^. Common monocyte progenitors (cMoPs) from bone marrow were gated on CD45^+^CD16/32^hi^ CD117^+^CD34^+^CD135^-^ and Ly6C^+^CD115^+^. The FACS data were analyzed by FlowJo software version 10.0.

### Western blotting

Total protein was extracted using RIPA lysis buffer (Beyotime Biotechnology, P0013). Approximately 30 μg of total protein was loaded per lane and separated by SDS-PAGE. Proteins were then transferred to PVDF membranes. After blocking with 5% skimmed milk for 1 h, membranes were incubated sequentially with primary antibodies (LOX-1: Cat No. 11837-1-AP, CD36: Cat No. ab133625, SR-A1: Cat No. MCA1322, SR-B11: Cat No. 21277-1-AP, ABCA1: Cat No. 26564-1-AP, ABCG1: Cat No. 13578-1-AP, LIPA: Cat No. 12956-1-AP, GAPDH: Cat No.GB15002, HSP90: Cat No.GB12284) and secondary antibodies (HRP-conjugated anti-rabbit IgG1: Cat No. 15004). Protein bands were finally visualized using the BeyoECL Plus chemiluminescence kit (Beyotime Biotechnology, P0018S).

### ELISA

Plasma LIPA protein levels were quantified using a commercially available ELISA kit (ELK Biotechnology, ELK7101) according to the manufacturer's instructions. Absorbance was measured using a BioTek® microplate reader.

### Statistical analysis

All data were presented as means ± SEM. GraphPad Prism software (version 8.0) was used for data analysis. Statistical significance was assessed by one-way ANOVA (use the Geisser-Greenhouse correction) and Tukey when three groups were compared. Significant differences between different groups were set at ∗*P <* 0.05, ∗∗*P <* 0.01, ∗∗∗*P <* 0.001and ∗∗∗∗*P <* 0.0001.

## Results

### *Lipa* deficiency results in splenomegaly and pro-myeloid differentiation in HFD-fed *Apoe*^*−/−*^ mice

**Fig. 1:**
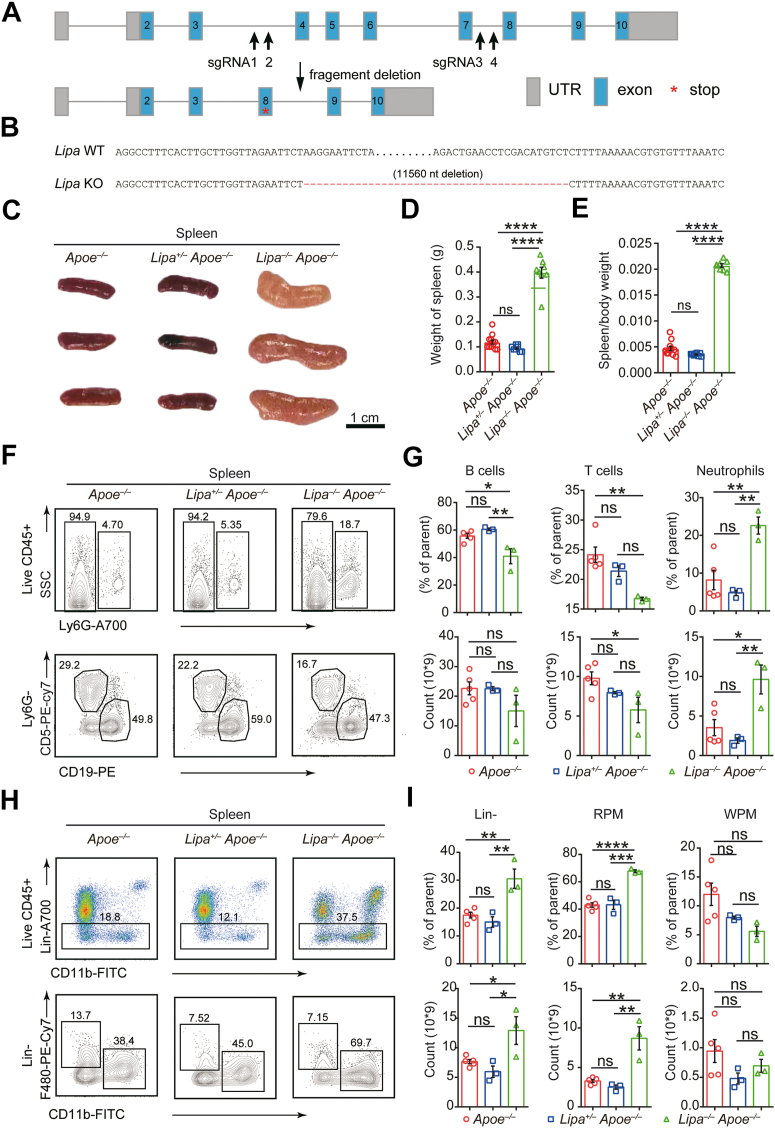
*Lipa* deficiency results in splenomegaly and increase of myeloid cells in *Apoe*^*−/−*^ mice. A: Schematic representation of the two guide RNA (sgRNA) sequences used to target the *Lipa gene*. B: DNA sequencing analysis showed the presence of the intended *Lipa* knockout mutation in F0 animals, and red dashes correspond to deleted. C: representative images of spleen in *Apoe*^−/−^, *Lipa*^+/−^*Apoe*^−/−^ and *Lipa*^−/−^*Apoe*^−/−^ mice on a high fat diet for 12 weeks. Scale bar = 1 cm. D and E: Quantification of spleen weight and spleen index in *Apoe*^−/−^, *Lipa*^+/−^*Apoe*^−/−^, *Lipa*^−/−^*Apoe*^−/−^ mice on a high fat diet for 12 weeks (n = 8 to 12). (F and G) Representative flow cytometric analysis and quantification of CD19^+^ B cells, CD5^+^ T cell, Ly6G^+^ neutrophils in spleen of *Apoe*^−/−^, *Lipa*^+/−^*Apoe*^−/−^ and *Lipa*^−/−^*Apoe*^−/−^ mice. (n = 3 to 5). H and I: Representative flow cytometric analysis and quantification of lin^-^ (CD3^-^CD19^-^Ly6G^-^NK1.1^-^), RPM (CD11b^+^F4/80^-^) and WPM (CD11b^-^F4/80^+^) cells in spleen of *Apoe*^−/−^, *Lipa*^+/−^*Apoe*^−/−^ and *Lipa*^−/−^*Apoe*^−/−^ mice. (n = 3 to 5). Data represent the mean ± SEM. ∗*P* < 0.05, ∗∗*P* < 0.01, ∗∗∗*P* < 0.001, ∗∗∗∗*P* < 0.0001, ns, non-significant.

To understand the role of *Lipa* in mice with hyperlipidemia, we performed *Lipa* knockout on the *Apoe*^*−/−*^ background by using CRISPR/Cas9 genome-editing tools. As described in the Material and Methods section, we targeted exons 4, 5, 6, and 7, which resulted in deletion of a large DNA segment of 11.5 kb in size on the C57BL/6 *Apoe*^*−/−*^ background (Fig. 1A, B). To model hyperlipidemia and atherosclerosis, we fed *Apoe*^*−/−*^, *Lipa*^*+/−*^ *Apoe*^*−/−*^, and *Lipa*^*−/−*^ *Apoe*^*−/−*^ mice a high-fat diet for 12 weeks. During the high-fat diet feeding, we observed that *Lipa*^*−/−*^ *Apoe*^*−/−*^ double knockout mice displayed progressive splenomegaly, with an average splenic weight more than three times that of *Apoe*^*−/−*^ mice. In contrast, the heterozygous *Lipa* knockout *Lipa*^*+/−*^ *Apoe*^*−/−*^ mice did not show any signs of splenomegaly (Fig. 1C, D). We further performed flow cytometric analyses to assess the impact of *Lipa* deficiency on immune cells in hyperlipidemic mice. To our surprise, we found that homozygous *Lipa* deficiency on an *Apoe*^*−/−*^ background markedly decreased the frequencies of T and B lymphocytes, whereas it markedly increased the frequencies of myeloid cells, including neutrophils and red pulp macrophages in the spleen (Fig. 1E–H). Taken together, our results show that the absence of *Lipa* contributes to alterations in myeloid and lymphoid cell populations in hyperlipidemic mice.

### Single-cell RNA-seq analysis identifies a proliferating macrophage population exclusively present in the spleen of *Lipa-*deficient hyperlipidemic mice

**Fig. 2:**
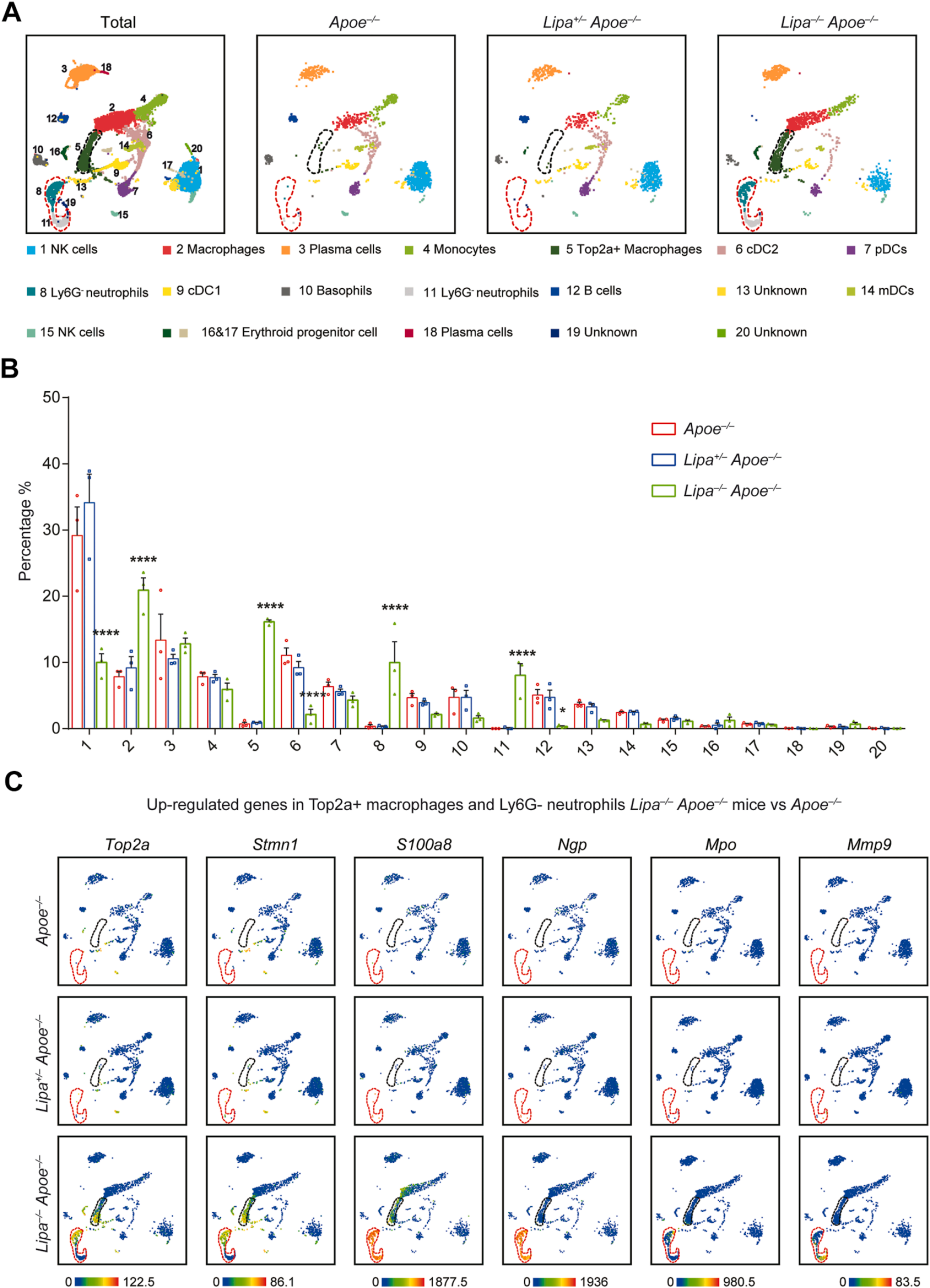
ScRNA-seq characterization of the spleen in *Apoe*^*−/−*^ mice and *Lipa*^−/−^*Apoe*^*−/−*^ mice fed with 12-weeks high-fat diet. A: UMAP plot showing 14 annotated cell types of CD45^+^CD5^-^CD19^-^ immune cells from spleen in *Apoe*^−/−^, *Lipa*^+/−^*Apoe*^−/−^ and *Lipa*^−/−^*Apoe*^−/−^ mice on a high fat diet for 12 weeks. Cluster 5 shown by polygonal gating of black dashed lines, and cluster 8 and cluster 11 shown by polygonal gating of red dashed lines. B: Histogram plot showing the proportion of different cell type in different group (n = 3). Data represent the mean ± SEM. ∗∗∗∗*P* < 0.0001. C: Heatmap statistic map showing 6 up-regulated genes in *Top2a*^*+*^ macrophages and *Ly6G*^*-*^ neutrophils in *Lipa*^−/−^*Apoe*^−/−^ versus. *Apoe*^−/−^ mice in three group, including *Top2a*, *Stmn1*, *S100a8*, *Ngp*, *Mpo* and *Mmp9*.

As we found markedly increased splenic myeloid cells, including neutrophils in *Lipa-deficient* mice by flow cytometry, we performed additional single-cell RNA-seq analysis to further characterize the impact of *Lipa* deficiency on the myeloid lineages in hyperlipidemic mice. To enrich and isolate myeloid cells, we employed a sorting technique to deplete T cells, Ly6G^+^ neutrophils and a majority of B cells. CD45^+^ CD5^-^CD19^-^ Ly6G^-^ splenocytes were sorted from the spleens of *Apoe*^*−/−*^ mice, *Lipa*^*+/−*^ *Apoe*^*−/−*^ mice, and *Lipa*^*−/−*^ *Apoe*^*−/−*^ mice that were fed a high-fat diet for 12 weeks. In this single-cell RNA-seq experiment, three animals for each genotype were labeled with unique sample tags, allowing for comparative and statistical analysis, as described in the Material and Methods section. Equal cell numbers from each genotype were pooled using barcode technology and processed together for library preparation and sequencing. Overall, 14,370 cells passing quality control from 9 mice were analyzed (supplemental Fig. S1). We used the Seurat 4.0 package to classify them into 20 clusters and annotate the immune cell identities based on the top 10 differentially expressed genes (supplemental Fig. S2, supplemental Table S2 and Fig. 2A). Strikingly, we found that the most dramatic increases in *Lipa*^*−/−*^ *Apoe*^*−/−*^ mice were in macrophages (Cluster 2) and proliferating macrophages (Cluster 5) which express marker genes related to cell cycle and proliferation, such as *Top2a* and *Stmn1*(Fig. 2B, C, and supplemental Fig. S2). Cluster 5, indicted by the black dashed polygonal, constituted 0.7% in *Apoe*^*−/−*^, 0.91% in *Lipa*^*+/−*^ *Apoe*^*−/−*^, and 16.3% in *Lipa*^*−/−*^ *Apoe*^*−/−*^ mice (Fig. 2B, C). Cluster 8 and Cluster 11, gated by a red dashed polygon, represent putative Ly6G^-^ neutrophils that express *Mpo*, *Ngp*, *Mmp9* and *S100a8* (Fig. 2C). These two clusters are only present in *Lipa*^*−/−*^ *Apoe*^*−/−*^ mice. As reported in previous studies, S100a8 is a neutrophil marker, and its expression can be found in splenic neutrophils that do not express Ly6G (26, 27). We also observed a significant decrease in Cluster 1 and Cluster 6, the populations corresponding to NK cells and type 2 conventional dendritic cells (Fig. 2B). Our single-cell RNA-seq data highlight the impact of the *Lipa* deficiency on promoting myeloid cell differentiation in the hyperlipidemic mice.

### Knockout of *Lipa*, whether homozygous or heterozygous, significantly attenuates atherosclerosis development in hyperlipidemic mice

**Fig. 3:**
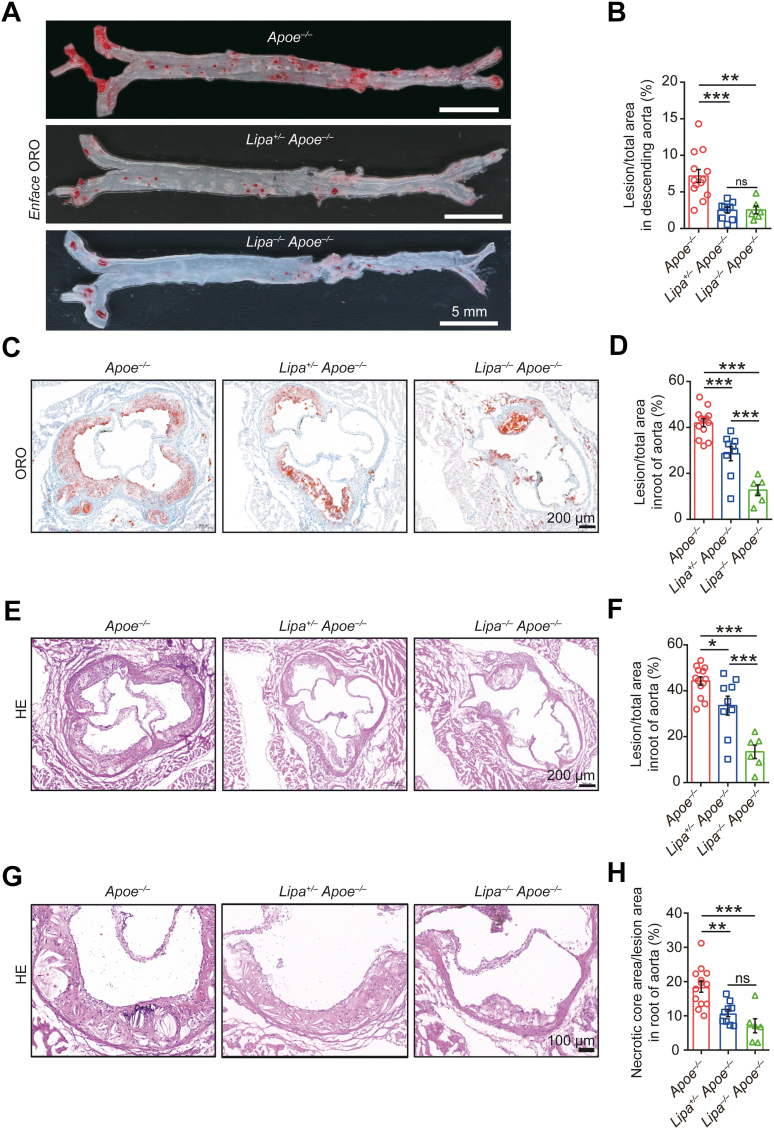
Depletion of *LIPA* reduces the atherosclerotic lesion formation in *Apoe–/– mice* A and. B: *Enface* ORO staining and quantification of lesion area in descending aorta in *Apoe*^−/−^, *Lipa*^+/−^*Apoe*^−/−^, *Lipa*^−/−^*Apoe*^−/−^ mice on a high-fat diet for 12 weeks (n = 6 to 13). C and D: ORO staining and quantification of lesion area in the root of the aorta among three groups. E and F: HE staining and quantification of lesion area in the root of aorta among three groups. G and H: HE staining and quantification of necrotic area in lesion area in *Apoe*^−/−^, *Lipa*^+/−^*Apoe*^−/−^, *Lipa*^−/−^*Apoe*^−/−^ mice on a high-fat diet for 12 weeks (n = 6 to 13). Scale bar = 100 μm. Data represent the mean ± SEM. ∗*P* < 0.05; ∗∗*P* < 0.01; ∗∗∗*P* < 0.001; ns, no statistical significance.

We performed further investigation to determine the potential impact of *Lipa* deficiency on atherosclerosis in hyperlipidemic mice. The aortic root and descending aorta from three groups of animals, including *Apoe*^*−/−*^ mice, *Lipa*^*+/−*^*Apoe*^*−/−*^ mice, and *Lipa*^*−/−*^ *Apoe*^*−/−*^ mice, were collected after 12-weeks high-fat diet feeding for pathological examination. *En face* ORO staining of the descending aorta showed that the atherosclerotic plaque in both *Lipa*^*+/−*^*Apoe*^*−/−*^ mice and *Lipa*^*−/−*^ *Apoe*^*−/−*^ mice was markedly reduced compared with that in *Apoe*^*−/−*^ mice (Fig. 3A). ORO and HE staining of the aortic root also revealed a significant decrease in atherosclerotic plaque formation in *Lipa*^*−/−*^ *Apoe*^*−/−*^ and *Lipa*^*+/−*^*Apoe*^*−/−*^ mice compared to *Apoe*^*−/−*^ mice (Fig. 3C–F). Subsequent pathological examination of aortic lesions revealed a significant reduction in necrotic core in *Lipa*^*−/−*^ *Apoe*^*−/−*^ and *Lipa*^*+/−*^*Apoe*^*−/−*^ mice compared to *Apoe*^*−/−*^ mice (Fig. 3G, H). Collectively, these results demonstrate that both homozygous and heterozygous *Lipa* deficiency significantly attenuates atherosclerosis.

### *Lipa* knockout significantly decreases accumulation of intra-plaque macrophage

**Fig. 4:**
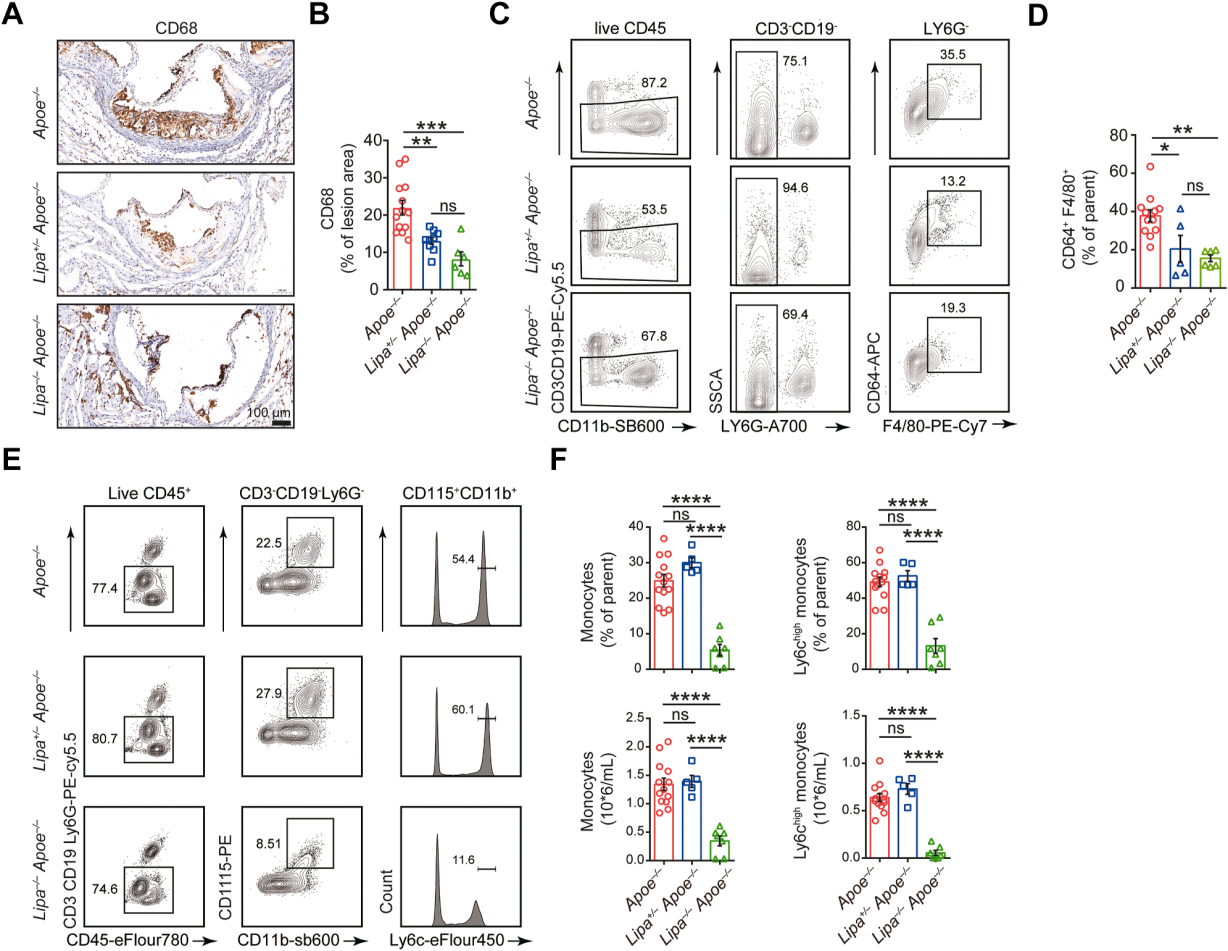
Loss of *Lipa* inhibits atherogenesis via lowering macrophage percentage in lesions. A and B: representative photomicrographs showing immunohistochemical staining of CD68 and quantitative analysis of positively stained lesion areas versus total lesion area for CD68 in lesion area in *Apoe*^−/−^, *Lipa*^+/−^*Apoe*^−/−^, *Lipa*^−/−^*Apoe*^−/−^ mice on a high-fat diet for 12 weeks (n = 6 to 13). Scale bar = 100 μm. C and D: representative flow cytometric analysis and quantification of macrophages (CD64^+^F4/80^+^) in the aorta of *Apoe*^−/−^, *Lipa*^+/−^*Apoe*^−/−^, and *Lipa*^−/−^*Apoe*^−/−^ mice. E and F: gating strategy used to analyze peripheral blood monocytes from *Apoe*^−/−^, *Lipa*^+/−^*Apoe*^−/−^, *Lipa*^−/−^*Apoe*^−/−^ mice on a high-fat diet for 12 weeks (n = 5 to 13). Monocytes were gated on CD45^+^CD3^-^CD19^-^Ly6G^-^ and CD11b^+^CD115^+^. Data represent the mean ± SEM. ∗*P* < 0.05; ∗∗*P* < 0.01; ∗∗∗*P* < 0.001; ns, no statistical significance.

The pathological analysis of homozygous and heterozygous *Lipa* knockout on the *Apoe*^*−/−*^ mouse background was intriguing. Since the changes in splenic myeloid cell phenotype, as analyzed by flow cytometry and single-cell RNA-seq, were observed only in the homozygous *Lipa*^*−/−*^ *Apoe*^*−/−*^ mice, not in the heterozygous *Lipa*^*+/−*^*Apoe*^*−/−*^ mice, which retain a functional allele of the *Lipa* gene. Therefore, we were interested in investigating whether changes in macrophages occurred within the atherosclerotic plaque of *Lipa*^*+/−*^*Apoe*^*−/−*^ mice with hyperlipidemia, using *Apoe*^*−/−*^ mice as controls. We conducted an analysis of macrophages in atherosclerotic plaques from *Lipa*^*−/−*^ *Apoe*^*−/−*^, *Lipa*^*+/−*^ *Apoe*^*−/−*^ and *Apoe*^*−/−*^ mice, after 12 weeks of high-fat diet feeding. Interestingly, immunohistochemical staining with an anti-CD68 antibody revealed a significant reduction in the accumulation of intraplaque macrophages in both *Lipa*^*−/−*^ *Apoe*^*−/−*^ mice and *Lipa*^*+/−*^ *Apoe*^*−/−*^ mice compared with *Apoe*^*−/−*^ controls (Fig. 4A, B). We also performed flow cytometric analysis of aortic macrophages by gating CD64^+^ F4/80^+^ cells from live cells expressing CD45 but negative for CD3, CD19, and Ly6G. We observed a significant decrease in the percentage of CD64^+^ F4/80^+^ macrophages in both *Lipa-*deficient groups (Fig. 4C, D). We hypothesized that the reduced accumulation of intraplaque macrophages may be associated with circulating monocytes. To investigate this, we analyzed Ly6C^high^ peripheral blood monocytes, known for their high foam cell formation capacity and atherosclerosis contribution (28). By gating CD11b and CD115 double-positive cells (29), we examined Ly6C^high^ monocytes in *Lipa*^*−/−*^ *Apoe*^*−/−*^, *Lipa*^*+/−*^ *Apoe*^*−/−*^, and *Apoe*^*−/−*^ mice. The results revealed that only homozygous *Lipa*^*−/−*^ *Apoe*^*−/−*^ mice exhibited decreased frequencies and absolute numbers of monocytes and Ly6C^high^ circulating monocytes during hyperlipemia (Fig. 4E, F). As peripheral blood monocytes originate from bone marrow precursors (30), we further analyzed monocyte developmental dynamics in the bone marrow of *Lipa*^*−/−*^ *Apoe*^*−/−*^ and *Apoe*^*−/−*^ mice by flow cytometry. Results demonstrated significant reductions in the total numbers of common myeloid progenitors (CMPs), granulocyte-monocyte progenitors (GMPs) and common monocyte progenitors (cMoPs) in *Lipa*^*−/−*^ *Apoe*^*−/−*^mice relative to *Apoe*^*−/−*^ control mice (supplemental Fig. S3).

**Fig. 5:**
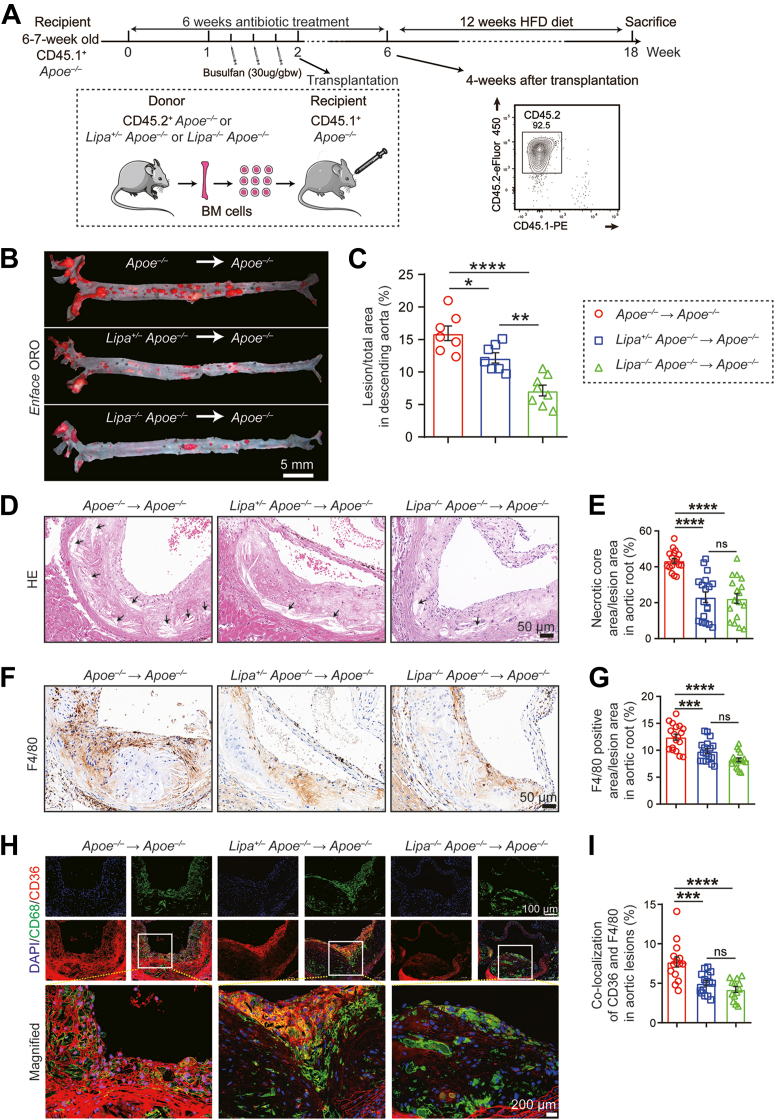
*Lipa* deficiency in bone marrow inhibits atherogenesis by reducing lesional macrophage. A: diagram of the bone marrow transplantation protocol. B and C: *Enface* ORO staining and quantification of lesion area in descending aorta in *Apoe*^−/−^ recipient mice reconstituted with bone marrow from *Apoe*^−/−^, or *Lipa*^+/−^*Apoe*^−/−^, or *Lipa*^−/−^*Apoe*^−/−^ donors (n = 7 to 8). Scale bar = 5 mm. D and E: Necrotic area quantification within lesions. 3 lesions per mice. Scale bar = 50 μm. F and G: representative immunohistochemistry for macrophage marker F4/80 and quantitative analysis of F4/80 positive areas versus total lesion areas. (n = 6 per group, 2–3 lesions per mice. Scale bar = 50 μm. H and I: representative immunofluorescence staining of CD68 (green) and CD36 (red) in aortic lesions, with quantitative analysis of CD68 and CD36 double positive areas (white arrows) as a percentage of total lesion areas (n = 6 per group, 2–3 lesions per mice). Scale bars, 100 μm in the original images and 200 μm in the magnified images. Data represent the mean ± SEM. ∗*P* < 0.05; ∗∗∗*P* < 0.001; ∗∗∗∗*P* < 0.0001; ns, no statistical significance.

To further confirm the mono-macrophages contribution of *Lipa* deficiency to atherosclerosis, we performed bone marrow transplantation using *Apoe*^*−/−*^, *Lipa*^*+/−*^ *Apoe*^*−/−*^ and *Lipa*^*−/−*^ *Apoe*^*−/−*^ mice that carry the CD45.2 congenic marker as donors, and CD45.1^+^ *Apoe*^*−/−*^ mice as recipients (Fig. 5A). Our data showed that body weight, lipid profile, and hepatic lipid accumulation were comparable among three recipients of groups after 12 weeks of high-fat diet (supplemental Fig. S4A–D). The *en face* ORO staining revealed that recipients of heterozygous bone marrow exhibited a modest reduction in plaque area, while those receiving homozygous bone marrow showed a marked decrease (Fig. 5B, C). HE staining further demonstrated that significant reduction in necrotic core area in both heterozygous and homozygous recipient groups (Fig. 5D, E). Interestingly, immunofluorescence staining results revealed the proportions of F4/80^+^ macrophages and CD36^+^ CD68^+^ macrophages in the aortic root were decreased in both heterozygous and homozygous recipient groups relative to controls. (Fig. 5F–I).

In summary, both homozygous *Lipa*^*−/−*^ *Apoe*^*−/−*^ mice and heterozygous *Lipa*^*+/−*^ *Apoe*^*−/−*^ mice exhibited reduced intra-plaque macrophage accumulation, but through distinct mechanisms. In homozygous mice, the reduction was driven by impaired monocyte development, reflected by decreased bone marrow progenitors and circulating Ly6C^high^ monocytes. In heterozygous mice, the effect was associated with reduced CD36 expression in macrophages, limiting foam cell formation.

### *Lipa* deficiency suppresses foam cell formation through downregulation of CD36

**Fig. 6:**
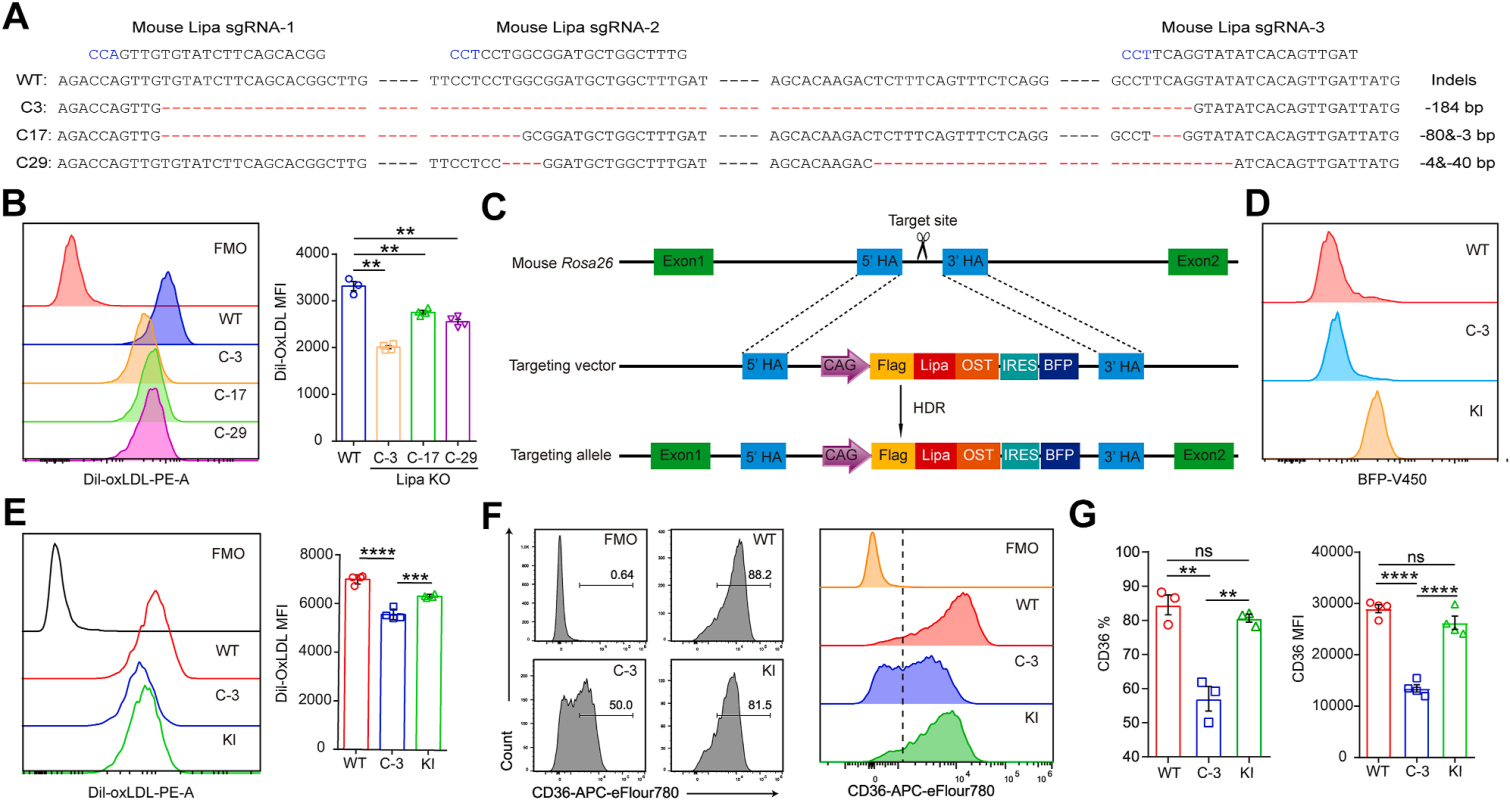
Genetic ablation of *Lipa* reduces and knock-in of *Lipa* rescues foam cell formation via regulation of CD3. A: Mutated sequences encompassing the site targeted by the CRISPR/Cas9 in the *Lipa*^−/−^ RAW264.7 cell clones C3, C17 and C29. The deletions observed in each mutant allele and the corresponding WT sequence are shown. Blue letters indicate the corresponding PAM sequences, and red dashes represent deleted nucleotides. B: DiI-OxLDL uptake was measured by flow cytometry as MFI using WT RAW264.7 cells and three *Lipa*^−/−^ RAW264.7 clones (denoted C3, C17 and C29). Histogram shows the mean MFI ± SEM of triplicate samples. C: Strategy for introducing the CAG-Flag-Lipa-OST-BFP cassette via knock-in (KI) in the mouse Rosa26 locus. CAG-Flag-Lipa-OST-BFP cassette containing ∼1 kb 5′ and 3′ homology arms (HA) targeted into the Rosa26 locus via homology-directed repair (HDR). The full-length Lipa open reading frame was fused at its 3′ end to an OST tag and placed under the control of the CAG hybrid promoter. The expression of Lipa can be monitored by BFP reporter which is coupled to the expression of Lipa-OST by a flag. D: Flow cytometry result shows the BFP reporter in WT and Lipa-OST KI macrophages. E: DiI-OxLDL uptake was measured by flow cytometry as MFI using WT RAW264.7 cells, *Lipa*^*−/−*^ RAW264.7 clone (denoted C-3) and Lipa-OST KI RAW264.7 clone. F and G: The percentage of CD36^+^ macrophages and CD36 MFI were measured by flow cytometry in WT RAW264.7 cells, *Lipa*^*−/−*^ RAW264.7 clone (denoted C-3) and Rosa26-Lipa-BFP; *Lipa*^*−/−*^ RAW264.7 (KI). Data represent the mean ± SEM. ∗∗*P* < 0.01; ∗∗∗*P* < 0.001; ∗∗∗∗*P* < 0.0001; ns, no statistical significance.

We observed a significant reduction in macrophages residing in atherosclerotic plaques in mice with both heterozygous and homozygous *Lipa* deficiency. To further investigate the impact of *Lipa* deficiency on macrophage function, we analyzed the foam cell-forming ability of *Lipa* deficiency. Foam cell formation is a crucial step in the development of lipid-laden macrophages, which contribute to the pathology of atherosclerotic plaques. First, we performed genetic deletion of *Lipa* gene in the murine RAW264.7 macrophages via CRISPR/Cas9. Guide RNAs specifically targeting *Lipa* (ENSMUST00000049572.14) was designed to disrupt exon 4, and sanger sequencing confirmed DNA sequence alteration resulting from CRISPR/Cas9 editing (supplemental Fig. S5 and Fig. 6A). Further functional assay using DiI-OxLDL showed that *Lipa* deficient macrophages exhibited significantly reduced lipid uptake (Fig. 6B). In parallel, we also generated a genetic complementation by knocking *Lipa* into the Rosa26 locus. We re-expressed *Lipa* in the *Lipa*-deficient RAW264.7 cells using an expression cassette permitting expression of *Lipa* under the control of the CAG promoter and coupled to a BFP reporter to facilitate isolation and identification of *Lipa* expressing cells (Fig. 6C). Knock-in of the cassette in mouse Rosa26 locus was achieved via homologous-dependent recombination (HDR) and validated by flow cytometry (Fig. 6D). Further functional assay showed that re-expression of *Lipa* restored lipid uptake in *Lipa* deficient macrophages (Fig. 6E). To investigate the mechanism underlying the reduced lipid uptake capacity in *Lipa*-deficient macrophages, we examined the expression of key lipid metabolism-related genes by qPCR and Western blot. qPCR analysis confirmed that *Lipa* mRNA was significantly reduced in *Lipa*^*−/−*^ (C-3) cells and restored in the knock-in (KI) cells. Among the scavenger receptors, CD36 was significantly downregulated upon *Lipa* deficiency, while *SR-A1* and *LOX-1* were upregulated. Regarding cholesterol efflux transporters, *ABCA1* was decreased and *SR-B1* was increased, whereas *ABCG1* showed no significant change in C-3 cells but was elevated in KI cells (supplemental Fig. S6). Notably, Western blot analysis revealed that only CD36 protein expression was significantly reduced in C-3 cells and rescued in KI cells, with no significant changes in SR-A1, LOX-1, SR-B1, ABCA1, or ABCG1 across the three cell lines (supplemental Fig. S7). Given the consistent downregulation of CD36 at the mRNA and protein levels, we next validated CD36 protein expression by flow cytometry. Consistent with the qPCR and Western blot results, *Lipa* deficiency led to a significant reduction in both the percentage of CD36^+^ macrophages and the mean fluorescence intensity (MFI) of CD36. Re-expression of *Lipa* restored the proportion of CD36^+^ cells and CD36 MFI to a level comparable to that of wild type controls (Fig. 6F, G). These data suggest that *Lipa* deficiency suppresses foam cell formation through downregulation of CD36.

**Fig. 7:**
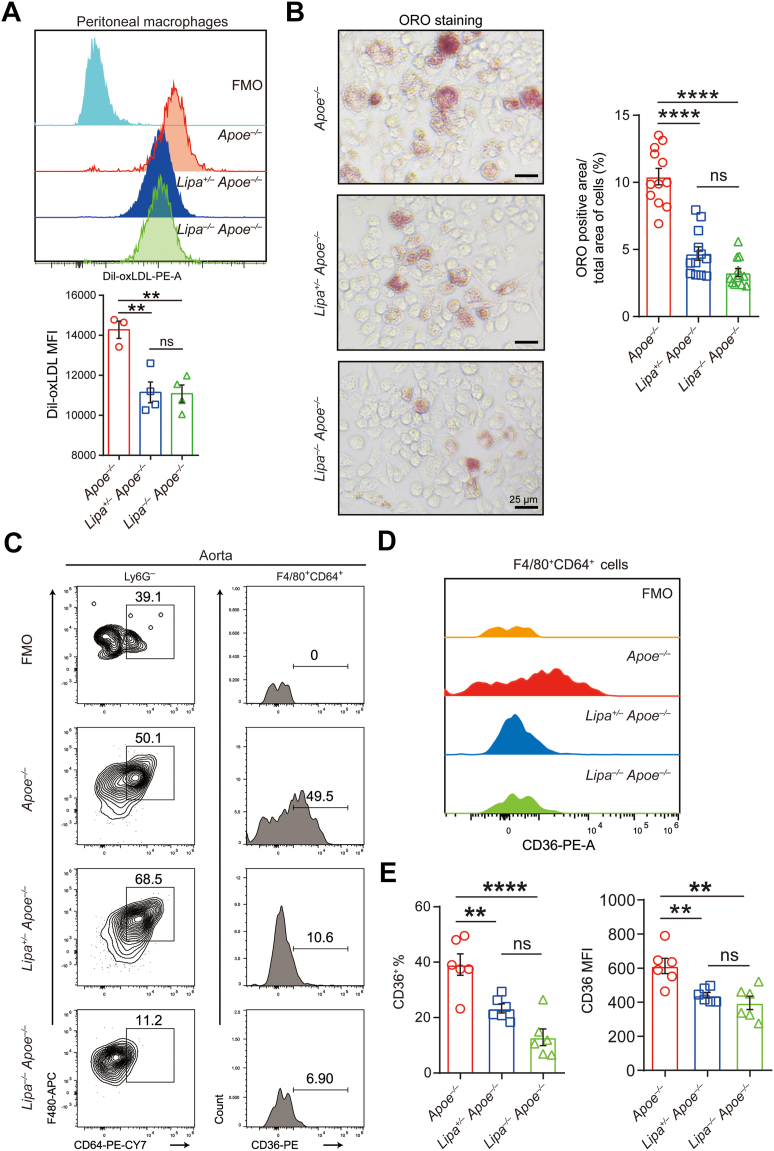
*Lipa* Deficiency reduces CD36-mediated Lipid Accumulation in Macrophages. A: DiI-OxLDL uptake was measured by flow cytometry as mean fluorescence intensity (MFI) using peritoneal macrophages from *Apoe*^−/−^, *Lipa*^+/−^*Apoe*^−/−^, *Lipa*^−/−^*Apoe*^−/−^ mice. Histogram shows the mean MFI ± SEM of triplicate samples. B: Representative images and statistical analysis of ORO-stained peritoneal macrophages of from *Apoe*^−/−^, *Lipa*^+/−^*Apoe*^−/−^, *Lipa*^−/−^*Apoe*^−/−^ mice (n = 12 regions from 3 mice, 4 regions per mice). The lipid droplet was stained in red color, scale bar = 25 μm. C–E: Representative flow cytometric analysis and quantification of CD36^+^ aortic macrophages (CD64^+^F4/80^+^) and CD36 MFI in *Apoe*^−/−^, *Lipa*^+/−^*Apoe*^−/−^, and *Lipa*^−/−^*Apoe*^−/−^ mice (n = 6). Data represent the mean ± SEM. ∗∗*P* < 0.01; ∗∗∗∗*P* < 0.0001; ns, no statistical significance.

To further investigate whether *Lipa* influences atherosclerosis development by modulating CD36-mediated foam cell formation, we first assessed lipid uptake capacity in peritoneal macrophages isolated from *Lipa*-deficient mice. Flow cytometry and ORO staining revealed that lipid uptake was significantly reduced in macrophages from both homozygous and heterozygous *Lipa*-deficient mice (Fig. 7A, B). We next examined CD36 expression in aortic macrophages from mice fed a high-fat diet for 12 weeks. Analysis of aortic CD64^+^F4/80^+^ macrophages showed that the percentage of CD36^+^ cells and CD36 MFI were both markedly decreased in mice with either heterozygous or homozygous *Lipa* deficiency mice (Fig. 7C–E).

Taken together, these in vitro and in vivo data demonstrate that *Lipa* deficiency inhibits atherosclerosis by impairing CD36-mediated foam cell formation.

### Lipid metabolism of heterozygous *Lipa*^*+/−*^*Apoe*^*−/−*^ is comparable to *Apoe*^*−/−*^ controls

**Fig. 8:**
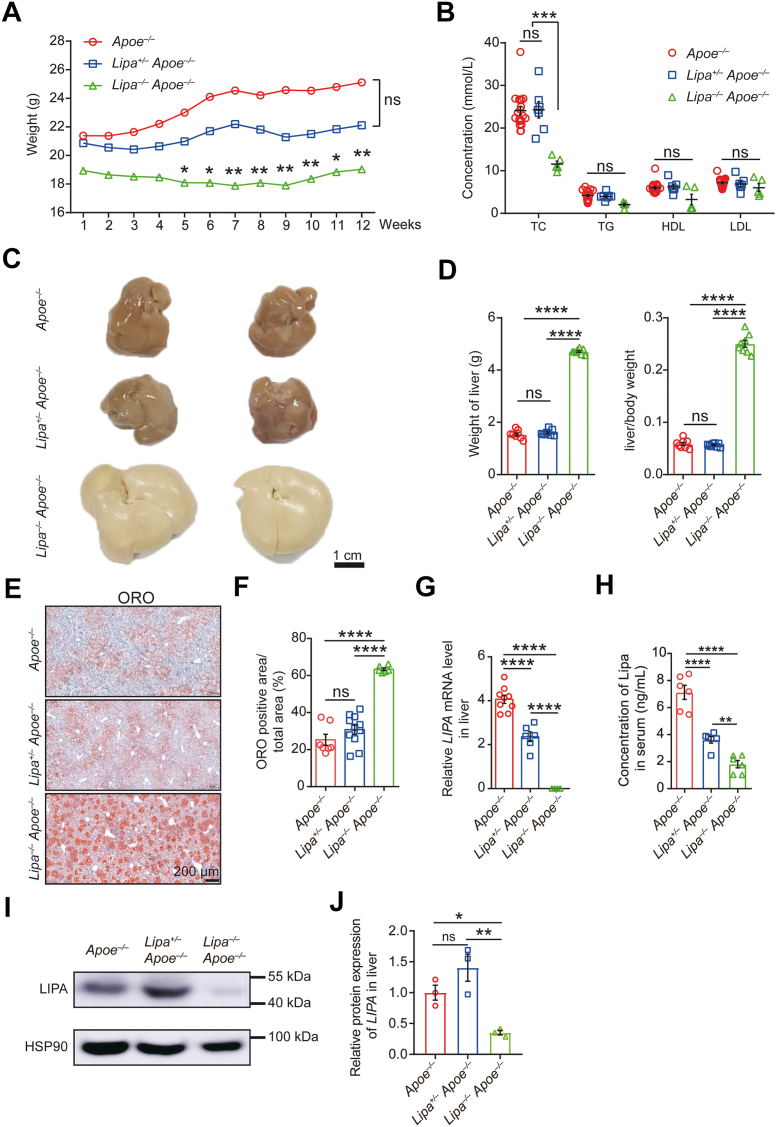
Lipid metabolism of homozygous *Lipa*^*−/−*^ Apoe^*−/−*^ is inhibited, but heterozygous *Lipa*^*+/−*^*Apoe*^*−/−*^ is unchanged compared with ApoE^*−/−*^ controls. A: Body weight of *Apoe*^−/−^, *Lipa*^+/−^*Apoe*^−/−^, and *Lipa*^−/−^*Apoe*^−/−^ mice on a high fat diet for 12 weeks (n = 6 to 10). B: Comparisons of serum lipid composition total serum cholesterol (TC), triglyceride (TG), low-density lipoprotein (LDL) and high-density lipoprotein (HDL) among three groups (n = 5 to 19). C and D: Representative images and quantification of liver weight and liver index in three groups (n = 8). Scale bar = 1 cm. E and F: ORO staining and quantification of ORO positive area in liver of three groups (n = 6 to 11). Scale bar = 100 μm. G: Relative mRNA levels of *Lipa* in the liver of three groups (n = 3 to 8). H: Concentration of LIPA in the blood of three groups (n = 6 per group). (I, J) Representative Western blot and quantification of *Lipa* protein expression levels in livers among three groups (n = 3 per group). Data represent the mean ± SEM. ∗*P* < 0.05; ∗∗*P* < 0.01; ∗∗∗*P* < 0.001; ∗∗∗∗*P* < 0.0001; ns, no statistical significance.

Since we observed that both homozygous and heterozygous *Lipa* deficiency similarly reduced atherosclerotic pathology and intraplaque macrophage accumulation, we sought to determine whether this phenomenon resulted from comparable metabolic alteration between homozygous and heterozygous *Lipa* deficiency mice. During the high-fat diet feeding period, we found that the body weight of heterozygous *Lipa*^*+/−*^ *Apoe*^*−/−*^ mice was similar with *Apoe*^*−/−*^ mice, but homozygous *Lipa*^*−/−*^ *Apoe*^*−/−*^ mouse were leaner and the body weight of *Lipa*^*−/−*^ *Apoe*^*−/−*^ was significantly lower than that of *Lipa*^*+/−*^ *Apoe*^*−/−*^ and *Apoe*^*−/−*^ mice (Fig. 8A). Further lipid profile examination showed that lipid profiles and the lipoprotein subclass composition in heterozygous *Lipa*^*+/−*^ *Apoe*^*−/−*^ mice were comparable with *Apoe*^*−/−*^ mice (Fig. 8B and supplemental Fig. S8), but the concentration of total cholesterol in homozygous *Lipa*^*−/−*^ *Apoe*^*−/−*^ mice was notably decreased when compared with that in *Lipa*^*+/−*^ *Apoe*^*−/−*^ and *Apoe*^*−/−*^ mice (Fig. 8B). As the liver is the main organ for lipid metabolism, we next examined liver weight and the lipid accumulation via ORO staining. The liver weight between heterozygous *Lipa*^*+/−*^ *Apoe*^*−/−*^ mice and *Apoe*^*−/−*^ mice were comparable, but homozygous *Lipa*^*−/−*^ *Apoe*^*−/−*^ mice displayed hepatomegaly, with an average hepatic weight more than three times that of *Apoe*^*−/−*^ mice (Fig. 8C, D). ORO staining results showed that the lipid accumulation in hepatocytes of heterozygous *Lipa*^*+/−*^ *Apoe*^*−/−*^ mice was comparable to *Apoe*^*−/−*^ mice but increased in the hepatocytes of homozygous *Lipa*^*−/−*^ *Apoe*^*−/−*^ mice (Fig. 8E, F). Further qPCR analysis of liver showed that the expression level of *Lipa* was significantly decreased in heterozygous mice, and no expression was detected in homozygous mice (Fig. 8G). The ELISA result showed that the concentration of LIPA was obviously reduced in both heterozygous and homozygous *Lipa* *deficiency* mice (Fig. 8H). But Western blot analysis revealed that LIPA protein expression was significantly reduced in homozygous mice, whereas no changes were observed in heterozygous mice (Fig. 8I, J).

To elucidate the mechanisms underlying divergent hepatic lipid phenotypes, normal metabolism in heterozygous mice versus profound steatosis in homozygous mice, we assessed hepatic expression of key regulators, including LOX-1, SR-A1, CD36, ABCA1, ABCG1, and SR-B1 (31). The Western blot results showed that homozygous mice significantly downregulated the lipid uptake receptors LOX-1 and CD36, as well as the cholesterol efflux transporters ABCA1 and SR-B1. In contrast, heterozygous mice exhibited selective downregulation of CD36 expression but compensatory ABCA1 upregulation (supplemental Fig. S9). This differential expression landscape underpins the observed phenotypic dichotomy.

Collectively, these results indicate that heterozygous mice maintained normal lipid metabolism, distinct from the profound dysregulation observed in homozygous mice, although both heterozygous and homozygous *Lipa* deficiency had identical reductions in atherosclerosis severity and intraplaque macrophage accumulation on *Apoe*^*−/−*^ background.

## Discussion

*LIPA* deficiency is a rare, recessive autosomal diseases caused by the mutation of *LIPA* gene (4, 32), and the prevalence is estimated at approximately 3.13–4.86 cases per million births (2, 33). Moreover, loss of function mutations in *LIPA* are linked to accelerated atherosclerosis (7, 8, 9, 34). However, *LIPA* was highly expressed in infiltrating macrophages in human diseased arteries (18). Here, we addressed these controversies of by identifying the novel functions of *LIPA* in the atherosclerosis development.

In this study, we established a novel strain of *Lipa* and *Apoe* double-knockout mice. Notably, homozygous *Lipa*^*−/−*^ *Apoe*^*−/−*^ mice exhibited normal developmental progression at birth and survived to adulthood (≥6 months) on a chow diet, but developed splenomegaly, and mortality after 12 weeks of high-fat feeding, recapitulating features of human CESD (35), as well as previous animal models (36, 37). scRNA sequencing data revealed a marked decrease in NK cells and cDC2s, along with a pronounced expansion of macrophages in homozygous *Lipa* knockout mice, likely contributing to splenic enlargement. Notably, proliferating macrophages, identified by high expression of proliferation marker genes *Top2a* (38, 39) and *Stmn1* (40, 41) in *S100a8*^*+*^ populations, and *Ly6G*^*-*^*S100a8*^*+*^ neutrophils (26, 27) were exclusively detected in the spleens of homozygous *Lipa* knockout mice. The expansion of red pulp macrophages and concomitant reduction of T and B cells within the spleen shares features with extramedullary hematopoiesis observed in other mouse models (42, 43). Interestingly, the increase in neutrophil counts in our *Lipa*^*−/−*^ *Apoe*^*−/−*^ mice contrasts with neutropenia commonly reported in other *Lipa*-knockout mice and human CESD patients (43, 44). This discrepancy may be attributable to the *Apoe*^*−/−*^ background (45), wherein profound hyperlipidemia and sustained inflammatory milieu in this model likely drive a compensatory extramedullary hematopoiesis response (44, 46), potentially overriding the cell-intrinsic impairment caused by *Lipa* deficiency. These findings suggest that *Lipa* deficiency-induced splenomegaly in a hyperlipidemic context results from disordered myeloid differentiation. Although classic extramedullary hematopoiesis is not typically characterized in human CESD patients, but rather by lipid-laden macrophage infiltration and splenic accumulation (35, 47), the concept of *Lipa* deficiency-induced myeloid dysplasia offers a plausible pathophysiological link. Further mechanistic studies into these processes will be essential to unraveling disease heterogeneity and advancing diagnostic and therapeutic strategies for CESD patients.

Although the changes in splenic myeloid cell phenotype between homozygous and heterozygous mice are different since heterozygous mice retain a functional allele of the *Lipa* gene, both heterozygous and homozygous *Lipa* knockout mice attenuate atherosclerotic lesion formation and the necrotic core in atherosclerotic lesions. Generally, macrophages derived from continuous recruitment and differentiation of peripheral blood monocytes (22, 48), especially the circulating Ly6C^high^ monocytes (49, 50, 51), are the main components in atherosclerotic lesions, and their internalization of modified lipids and cell debris leads to the buildup of a necrotic core and accelerates the development of atherosclerosis (52, 53). Moreover, a previous study also reported that *Lipa* cardiovascular risk alleles are expression quantitative trait loci for higher *Lipa* mRNA in monocytes and macrophages (7, 11, 54). Therefore, we next investigated the changes in monocytes and Ly6C^high^ monocytes in the blood and macrophages in the atherosclerotic plaque of *Lipa*^*+/−*^ *Apoe*^*−/−*^ mice with hyperlipidemia. The IHC experiment and flow cytometry experiment indicated that the percentage of macrophages in aorta was decreased in both heterozygous and homozygous *Lipa* deficiency mice. Both the percentage and absolute numbers of monocytes and Ly6C^high^ monocytes in peripheral blood were significantly reduced in homozygous mice. As established, peripheral monocytes are derived from bone marrow precursors via the CMPs→GMPs→cMoPs developmental cascade (30, 55). We observed concomitant decreases in CMPs, GMPs, and cMoPs populations in homozygous mice, indicating that *Lipa* deficiency attenuates atherosclerosis development primarily through impairment of bone marrow monopoiesis. Intriguingly, heterozygous mice maintained normal circulating Ly6C^high^ monocyte levels compared with *Apoe*^*−/−*^ mice. However, primary peritoneal macrophages from both homozygous and heterozygous mice exhibited a notable decrease in lipid uptake, demonstrating that impaired foam cell formation from macrophages represents a key complementary mechanism in athero-protection of *Lipa* deficiency. To further delineate the hematopoietic contribution of *Lipa* *deficiency* to atherosclerosis, we conducted a bone marrow transplantation experiment. Strikingly, *Apoe*^*−/−*^ mice transplanted with bone marrow cells from heterozygous or homozygous *Lipa* deficiency mice showed reduced aortic lesions, a smaller necrotic core and decreased macrophage accumulation. Consequently, the attenuated atherosclerosis observed in these mice is likely due not only to decreased accumulation of monocyte-derived macrophages into plaques but also to a systemic reduction in circulating monocytes available for infiltration.

Given the critical role of macrophages in *Lipa* deficiency-induced atherosclerosis alleviation, we established *Lipa* knockout and knock-in RAW264.7 macrophages via CRISPR/Cas9 technology. Bone marrow transplantation experiments revealed that *Apoe*^*−/−*^ mice receiving bone marrow cells from either heterozygous *Lipa*^*+/−*^ *Apoe*^*−/−*^ or homozygous *Lipa*^*−/−*^ *Apoe*^*−/−*^ mice exhibited reduced CD36 expression in aortic macrophages within atherosclerotic lesions. To further dissect the underlying mechanism, we employed *Lipa*^*−/−*^ and *Lipa*-rescued (KI) RAW264.7 macrophage cell lines. qPCR and Western blot analyses revealed that, among all scavenger receptors and cholesterol transporters examined, CD36 was the only molecule consistently downregulated at both the mRNA and protein levels in *Lipa*^*−/−*^ cells, with expression restored in KI cells. Although some transcriptional changes were observed for *SR-A1*, *LOX-1*, *ABCA1*, *ABCG1*, and *SR-B1*, these did not translate into corresponding protein alterations, suggesting they are unlikely to contribute functionally.

Given the well-established role of CD36 in mediating oxidized LDL uptake and foam cell formation (56, 57), our findings indicate that *Lipa* deficiency suppresses macrophage foam cell formation primarily through downregulation of CD36, rather than via other scavenger receptors or cholesterol transporters. This is corroborated by our in vivo data, which showed impaired lipid uptake, reduced CD36^+^ macrophage proportion, and decreased CD36 MFI within aortic plaques in *Lipa*-deficient mice. Collectively, these results confirm that *Lipa* deficiency attenuates macrophage-derived foam cell formation via CD36 downregulation.

*Lipa* deficiency leads to substantial cholesterol accumulation in the liver and spleen, yet paradoxically suppresses CD36 expression and foam cell formation specifically in aortic macrophages, suggesting tissue-specific heterogeneity in macrophage responses. We hypothesize that distinct metabolic and inflammatory microenvironments differentially modulate the impact of *Lipa* loss on macrophage function. Systemic lipid overload may alter monocyte-macrophage lineage programming, leading to CD36 downregulation that becomes most apparent in the context of atherosclerosis. This notion aligns with the established concept of macrophage heterogeneity across tissues, driven by local cues such as lipid composition, cytokine milieu, and cellular interactions. Notably, while *Lipa* deficiency causes profound lipid accumulation in the liver and spleen, the aortic microenvironment marked by sustained hyperlipidemia and inflammation may uniquely sensitize plaque macrophages to CD36 dysregulation. Alternatively, impaired monocyte development in the bone marrow of homozygous *Lipa*^*−/−*^ mice may generate a myeloid cell population with intrinsically altered scavenger receptor expression that manifests preferentially under inflammatory conditions such as atherosclerotic conditions.

*Lipa* deficiency patients commonly have a higher risk of cardiovascular disease due to the high levels of TC, TG, LDL and low levels of HDL in serum (58, 59, 60). But cardiovascular risk alleles are not associated with altered plasma lipids, liver traits, or reduced expression of *Lipa* in the liver (10, 61, 62). Our data reveal a genotype-dependent dichotomy in systemic and hepatic metabolism. Heterozygous *Lipa* knockout mice maintained body weight, serum lipids, and hepatic indices comparable to *Apoe*^*−/−*^ controls. While homozygous *Lipa* knockout mice exhibited reduced body weight, concentration of TC, and hepatic index. ORO staining of the liver also showed similar lipid accumulation between heterozygous *Lipa* knockout mice and control *Apoe*^*−/−*^ mice, but elevated hepatic lipid accumulation specifically in homozygous *Lipa* knockout mice. Further hepatic mechanistic analysis revealed that cholesterol efflux transporters ABCA1 and SR-B1 were significantly decreased in *Lipa*^−/−^ *Apoe*^−/−^ mice, possibly contributing to the lipid accumulation in hepatic cells. While ABCA1 was significantly increased in *Lipa*^+/−^ *Apoe*^−/−^ mice, which may restore lipid metabolism to normal levels. More importantly, bone marrow transplantation into *Apoe*^−/−^ recipients demonstrated that both body weight and lipid profiles were unchanged, regardless of donor genotype. These data illustrate that *Lipa* deficiency does not regulate atherosclerosis development through systemic lipid metabolism.

In summary, our data suggest that *Lipa* deficiency leads to splenomegaly due to an increase in neutrophils and macrophages in the spleen, and attenuates atherosclerosis development via disruption of monocyte development (homozygous-specific) and suppression of CD36-mediated macrophage-derived foam cell formation. To further dissect the underlying mechanisms, future studies using scRNA-seq across multiple tissues, including bone marrow, spleen, liver, and aorta, will help define the tissue-specific transcriptional landscape of macrophages in the context of *Lipa* deficiency.

## Data availability

The authors confirm that the data supporting the findings of this study are available within the article.

## Supplemental data

This article contains supplemental data.

## Conflict of interest

The authors declare that they have no conflicts of interest with the contents of this article.

## Figures and Tables

**sFigure 1:**
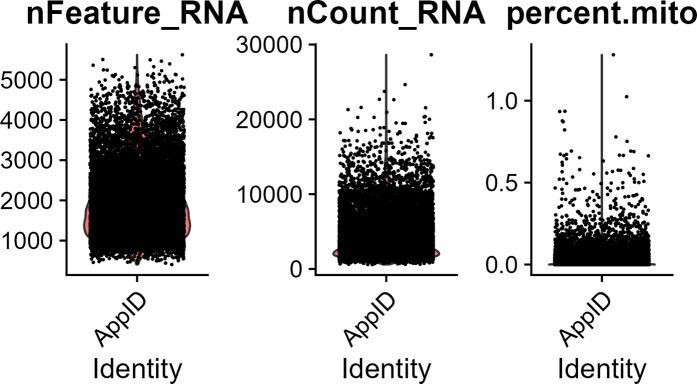


**sFigure 2:**
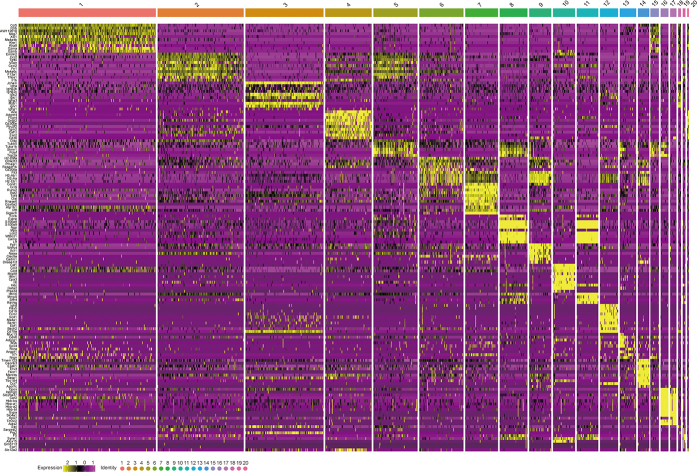


**sFigure 3:**
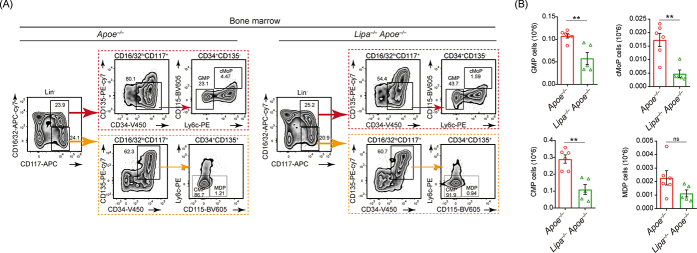


**sFigure 4:**
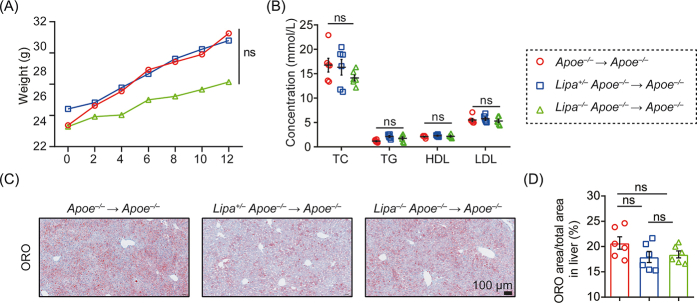


**sFigure 5:**
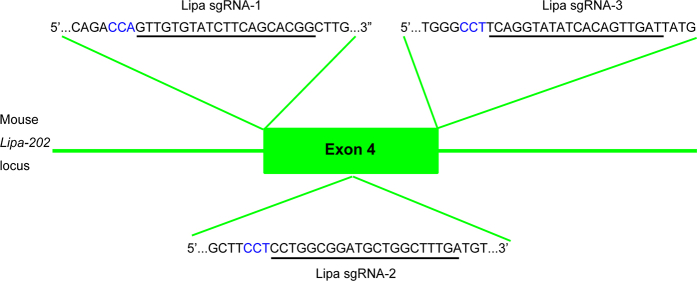


**sFigure 6:**
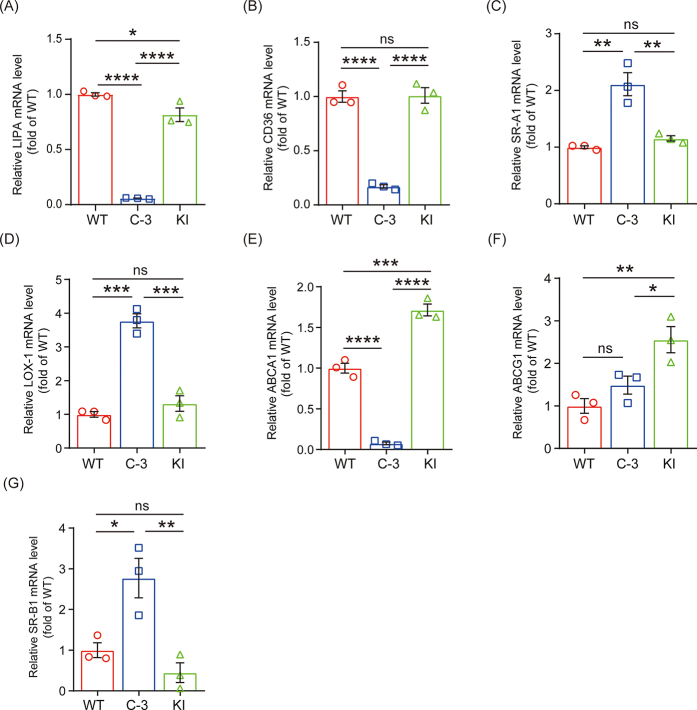


**sFigure 7:**
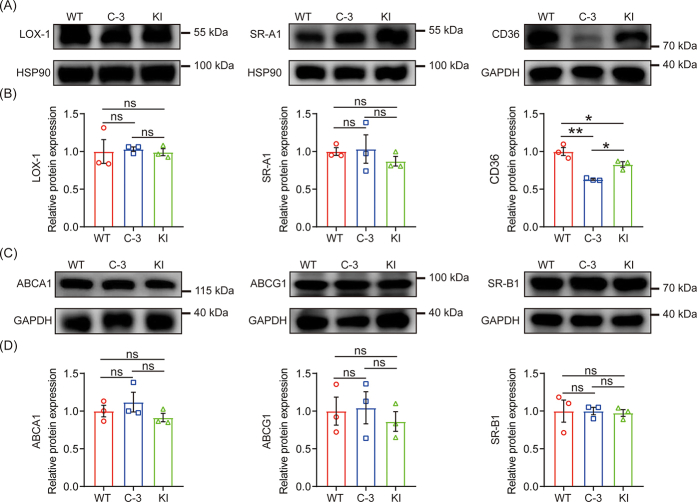


**sFigure 8:**
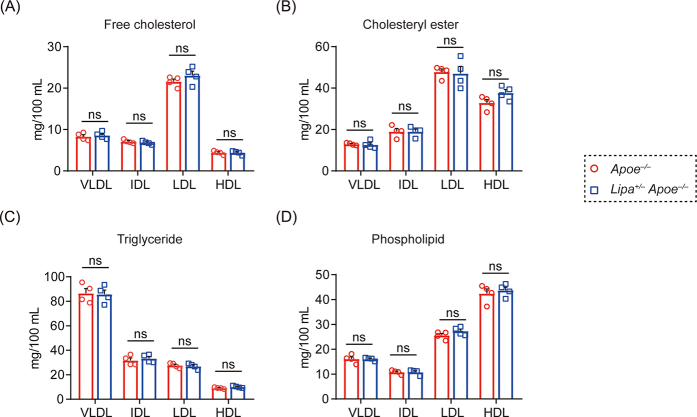


**sFigure 9:**